# CtChi19: a valuable gene for improving resistance to Botrytis cinerea and Alternaria alternata in Carthamus tinctorius

**DOI:** 10.3389/fpls.2026.1797378

**Published:** 2026-04-24

**Authors:** Kang Ma, Xiaoyan Wang, Kangjun Fan, Kehui Zhang, Lu Lv, Zhaojun Wei, Jiao Liu, Hong Liu, Jian Wei, Rui Qin

**Affiliations:** 1 Hubei Provincial Key Laboratory for Protection and Application of Special Plant Germplasm in Wuling Area of China, College of Life Sciences, South-Central Minzu University, Wuhan, China; 2 School of Biological Science and Engineering, North Minzu University, Yinchuan, China; 3 Institute for Safflower Industry Research of Shihezi University, Shihezi University, Shihezi, China

**Keywords:** Carthamus tinctorius, chitinase, fungal resistance, plant immunity, plant pathogen interaction

## Abstract

Safflower (*Carthamus tinctorius* L.) is an economically important crop with both medicinal and oil-producing values, However, its production is severely threatened by fungal diseases, including those caused by *Alternaria alternata* and other pathogenic fungi. To date, molecular investigations into the disease resistance mechanisms of safflower remain largely limited. In this study, proteomic analysis was performed on safflower following infection by *A. alternata*, identifying 490 and 921 differentially expressed proteins (DEPs) at 48 hours post-infection (hpi) and 72 hpi, respectively, Among these DEPs, a large number of candidate defense-related proteins were detected, including CtChi19. Meanwhile, by integrating previously published transcriptomic and proteomic data of safflower at the early stage of *Botrytis cinerea* infection, a set of candidate genes closely associated with defense responses was screened using weighted gene co-expression network analysis (WGCNA). Emphasis was placed on the hub gene *CtChi19*, which encodes a chitinase belonging to glycoside hydrolase family 19 (GH19). Subcellular localization assays showed that CtChi19 was localized in the cell wall and extracellular space. Furthermore, stable overexpression of *CtChi19* in safflower significantly enhanced the resistance of transgenic plants to both *B. cinerea* and *A. alternata*. Assays of chitinase and β-1,3-glucanase activities demonstrated that the improved disease resistance of OE-*CtChi19* transgenic plants was directly associated with a significant increase in chitinase and β-1,3-glucanase activities. Collectively, the candidate genes identified in this study provide valuable genetic resources and theoretical support for molecular breeding aimed at developing disease-resistant safflower varieties. These findings also offer novel insights for further dissecting the antifungal immune mechanisms in safflower.

## 1 Introduction

Safflower (*Carthamus tinctorius* L., 2*n* = 2*x* = 24), belonging to the Asteraceae family, is an economically important crop with dual medicinal and oil-bearing properties. It boasts a long cultivation history and is widely cultivated worldwide owing to its strong adaptability to barren, arid and high-temperature environments, as well as its diverse economic utilities (Wu et al., 2021b; Liu et al., 2025). Its core economic values are mainly embodied in the achenes and florets: the achenes are abundant in unsaturated fatty acids including linoleic acid and oleic acid, and the extracted safflower seed oil exerts notable health-promoting effects against cardiovascular and cerebrovascular diseases (Furuya et al., 1987; den Hartigh, 2019; Fan et al., 2023); the tubular florets contain hydroxy safflor yellow A (HSYA), a unique bioactive component with antioxidant, anti-inflammatory, anticoagulant, hypoglycemic and cardioprotective effects, which has been clinically used as a prescription drug for the treatment of coronary heart disease and angina pectoris in China (Furuya et al., 1987; Yan et al., 2022; Yin et al., 2024; Wang et al., 2025; Xi et al., 2025).

As an economically valuable crop, safflower is highly vulnerable to phytopathogen infection, especially during the mid-to-late growth stages. Foliar diseases are particularly severe in rainy regions from the late bud stage to the near-maturity stage (Kolte, 2018). Globally, a total of 57 pathogenic species have been reported to infect safflower, including 40 fungi, 2 bacteria, 14 viruses and 1 mycoplasma. Among them, *B. cinerea* can cause foliar diseases (Kolte, 2018, Kumar, 2021), and *Alternaria* leaf spot caused by *Alternaria carthami* ranks among the most destructive diseases affecting safflower. This disease has been reported in all safflower-producing countries worldwide and can cause yield losses of up to 25–60% in India (Anand and Kapoor, 2018). Fungal diseases generally lead to significant yield reduction and quality deterioration of safflower (Víchová et al., 2011; Singh et al., 2019). To date, studies on safflower diseases have predominantly focused on field investigations and pathogen identification, whereas molecular research on the mechanisms underlying safflower disease resistance remains largely limited. This situation not only restricts the in-depth dissection of safflower immune mechanisms but also impedes the breeding of disease-resistant safflower varieties.

This study aimed to elucidate the disease resistance mechanism of safflower and identify disease resistance genes for disease-resistant breeding. Tandem mass tags (TMT) labeling proteomics analysis was conducted on safflower infected by *A. alternata* to screen for candidate defense related proteins, and a set of such proteins were identified, including PR1, PR5, PR10, chitinase, LTPs (Lipid Transfer Protein), Pectin methylesterase, Catalase, LRRs (leucine-rich repeat protein) and phospholipase.

Among these identified proteins, chitinase is a vital antifungal component in plant innate immunity: as one of the most important antifungal proteins in plants, chitinase belongs to the PR-3, PR-4, PR-8 and PR-11 subfamilies of the pathogenesis-related (PR) protein family (Stintzi et al., 1993; Han and Schneiter, 2024). As a hydrolase that catalyzes the hydrolysis of chitin—a core component of fungal cell walls consisting of a polymer of N-acetylglucosamine linked by β-1,4-glycosidic bonds—it occupies a vital position in the plant defense system (Punja and Zhang, 1993; Sánchez-Vallet et al., 2015). Chitinases serve dual defensive functions. On the one hand, they directly degrade the cell walls of invading pathogenic fungi, thereby suppressing hyphal growth and spore germination and even inducing pathogen lysis and death (Sels et al., 2008); On the other hand, chitinases release soluble chitin oligosaccharide fragments (e.g., CO4–CO8) during the hydrolysis process. These fragments are recognized as non-self signals (i.e., damage-associated molecular patterns, DAMPs) by pattern recognition receptors (PRRs) on the plant cell membrane, such as CEBiP (chitin-elicitor binding protein) and CERK1 (chitin-elicitor receptor kinase-1) in rice, and LYM1 and LYM3 in Arabidopsis (Kaku et al., 2006; Miya et al., 2007; Hayafune et al., 2014), which in turn triggers a cascade of systemic defense responses including reactive oxygen species (ROS) burst, mitogen-activated protein kinase (MAPK) cascade activation, transcriptional upregulation of defense-related genes and lignin deposition (Sánchez-Vallet et al., 2015). In addition, the expression of plant chitinase is strongly induced by various biotic and abiotic stress signals such as pathogenic infection, mechanical wounding, ethylene and methyl jasmonate (Sels et al., 2008; Tang et al., 2017). its disease-resistant capacity has been well verified by transgenic studies in a wide range of plant species including: peanut (Rohini and Sankara Rao, 2001; Iqbal et al., 2012), cucumber (Tabei et al., 1998), strawberry (Vellicce et al., 2006), apple (Wang et al., 2021a), pepper (Ali et al., 2020), soybean (Yang et al., 2020), rice (Richa et al., 2017; Anwaar et al., 2024), as well as tobacco and maize (Liu et al., 2020).

In this study, proteomic analysis was performed on safflower following *A. alternat*a infection. By integrating previously published transcriptomic and proteomic datasets from safflower during the early stage of B. cinerea infection (12–72 hpi) (Ma et al., 2026), coupled with weighted gene co-expression network analysis (WGCNA), a chitinase-encoding gene designated *CtChi19* located on chromosome 12, was identified as a hub gene mediating the safflower defense response to fungal invasion. Subcellular localization assays revealed that the CtChi19 protein resides in the extracellular space. Furthermore, functional validation via stable overexpression of *CtChi19* in safflower confirmed that this gene significantly enhances host resistance to both *B. cinerea* and *A. alternata*. Assays of chitinase and β-1,3-glucanase activities indicated that the strengthened disease resistance is directly correlated with elevated chitinase activity. Collectively, this study expands the repertoire of candidate defense genes for dissecting the antifungal immune mechanisms in safflower, and provides valuable theoretical support for germplasm resource screening and resistance breeding in safflower improvement.

## 2 Materials and methods

### 2.1 Cultivation of plant materials

In this experiment, safflower cultivar YH-7 was used as the material for stable genetic transformation, and *Nicotiana benthamiana* was selected as the material for transient expression assays.

The genome-sequenced safflower cultivar Anhui-1 was employed as the primary experimental material. Seed pretreatment was conducted as follows: seeds were rinsed with sterile water, soaked for 24 h to promote germination, and then individually sown in pots filled with a mixed substrate of vermiculite, perlite and nutrient soil at a 1:1:1 volume ratio.

Plants were cultivated under a long-day photoperiod to regulate their growth cycle. After sowing, seedlings were maintained in a long-day growth chamber (16 h light/8 h dark, 22 °C) until the flowering stage (approximately 8–10 weeks), at which point they were used for genetic transformation. Transgenic safflower plants were subsequently grown under the same long-day conditions for an additional 4–6 weeks, and fungal inoculation assays were performed once the plants reached the appropriate growth stage.

*Nicotiana benthamiana* was used as the auxiliary experimental material for transient expression validation. Its seeds were sown directly in cultivation pots with a mixed substrate of vermiculite and nutrient soil (1:1, v/v) without sterile pretreatment. Ten days after sowing, uniformly growing seedlings were transplanted individually into new cultivation pots containing a mixed substrate of vermiculite, perlite and nutrient soil (1:1:1, v/v). After transplantation, the seedlings were transferred to a short-day growth chamber (12 h light/12 h dark, 22 °C) and cultured for approximately 4–5 weeks, and transient expression assays were conducted when the plants attained the growth status required for the experiments.

### 2.2 Cultivation of fungal and infection assays

*A. alternata* strain (isolated from safflower planting areas in Xinjiang and preserved in the Laboratory of College of Life Sciences, South-Central Minzu University) was cultured on potato dextrose agar (PDA) plates at 28 °C for 4–5 weeks. Spores were harvested and resuspended in a 2% glucose solution, and the spore concentration was adjusted to 1×10^5^ spores mL^-^¹. Safflower plants at the 5–6-week growth stage were pre-moisturized for 12 h, after which the 1×10^5^ spores mL^-^¹ spore suspension was uniformly spotted onto the leaf surfaces of transgenic and wild-type (WT) plants (10 μL per drop). For the control group, 2% glucose solution was used instead of the spore suspension. All plants were incubated in the dark for 12 h followed by moisturized cultivation for 48 h, and then transferred to a normal short-day culture condition (12 h light/12 h dark, 22 °C). Leaves were sampled at 48 and 72 hours post-inoculation (hpi), immediately frozen in liquid nitrogen, and stored for proteomic mass spectrometry analysis.

*A. alternata* strain and *B. cinerea* strain B05.10 were each cultured on PDA plates at 28 °C for 4–5 weeks until the mycelia overgrew the plates. Mycelial discs with uniform size were punched out from the plates using a cork borer. Safflower plants at the 5–6-week growth stage were pre-moisturized for 12 h, after which the uniform-sized mycelial discs were attached to the leaf surfaces of the plants. All plants were incubated in the dark for 12 h followed by moisturized cultivation for 48 h, and then transferred to a normal short-day culture condition (12 h light/12 h dark, 22 °C).

### 2.3 Proteomics and data analysis

In this study, safflower plants were inoculated with *A. alternata*, and mixed leaf samples were collected from 10 individual plants at two time points (48 hpi and 72 hpi) post-inoculation. A control group (treated with 5% glucose solution instead of spore suspension) was set up in parallel, with mixed leaf samples collected at the corresponding time points for subsequent proteomic analysis.

Protein extraction was performed using SDT lysis buffer (4% SDS, 100 mM Tris-HCl, pH 7.6), and the protein concentration was determined via the BCA assay. Trypsin digestion was carried out following the FASP protocol (Wiśniewski et al., 2009): trypsin was added at a mass ratio of 1:50 (trypsin:protein) and incubated at 37 °C for 15–18 h, after which the peptide fragments in the filtrate were collected. The peptides were desalted using a C18 solid-phase extraction (SPE) cartridge (Empore™ SPE Cartridges C18), concentrated under vacuum, and reconstituted in 40 μL of 0.1% formic acid solution; the peptide content was determined by measuring the ultraviolet absorbance at 280 nm. For each sample, 100 μg of peptides were labeled with TMT reagents in accordance with the manufacturer’s instructions from Thermo Scientific. All experiments were performed with three biological replicates.

The labeled peptides were analyzed using an Easy nLC system (Proxeon Biosystems, USA) coupled with a Q Exactive mass spectrometer (Thermo Fisher Scientific Co., Ltd, USA). Samples were first loaded onto an Acclaim epMap100 trap column, then separated on an Easy Column reversed-phase analytical column (75 μm × 10 cm, 3 μm C18 packing material) at a flow rate of 300 nL/min, with linear gradient elution performed using 0.1% formic acid in water (Buffer A) and 84% acetonitrile/0.1% formic acid (Buffer B). The mass spectrometer was operated in positive ion mode with data-dependent acquisition (Top20): the full scan range was set at m/z 300–1800 with a resolution of 60,000; precursor ions were fragmented via HCD (normalized collision energy of 30 eV), with an MS/MS resolution of 15,000, an isolation window of 1.5 m/z, an AGC target of 1e6, a maximum injection time of 50 ms, and a dynamic exclusion duration of 30 s.

Raw mass spectrometry data were searched against the safflower reference genome (https://www.scuec.edu.cn/safflower/) for protein identification and quantification using the MASCOT search engine (Matrix Science, London, UK; version 2.2) embedded in Proteome Discoverer 2.4. The false discovery rate (FDR) for identified peptides was set to ≤ 0.01, with a mass tolerance of ±20 ppm. All peptide ratios were normalized by the median method (to set the median protein ratio to 1). Differentially expressed proteins (DEPs) were defined as those with a fold change ≥1.2 or ≤0.83 and a *p*-value < 0.05 (Student’s t-test).

Functional annotation of DEPs was conducted based on the Gene Ontology (GO) database (Ashburner et al., 2000; Aleksander et al., 2023). The expression patterns of DEPs were visualized as heatmaps generated using TBtools (Chen et al., 2023).

### 2.4 WGCNA analysis and GO enrichment

Weighted gene co-expression network analysis (WGCNA) was performed on the proteomic data to screen for the hub protein CtChi19 in the highly correlated module. With CtChi19 as the core node, a co-expression network was constructed using Cytoscape (Shannon et al., 2003). Specifically, WGCNA was performed on the integrated proteomic datasets, which included the safflower proteomic profiles at 48 and 72 hours post-inoculation (hpi) with *A. alternat*a generated in this study, as well as the previously published safflower proteomic data at 48 and 72 hpi following *B. cinerea* inoculation (Langfelder and Horvath, 2008; Ma et al., 2026). The WGCNA-shinyApp plugin on the TBtools platform was used to divide the samples into treatment and control groups for module identification (Wang et al., 2024). In detail, the protein abundance data were subjected to an initial filter with the parameters of sample percentage = 0.9 and expression cutoff = 0.1. After filtering (removing all features with an expression value of less than 0.1 in more than 90% of the samples), a total of 6,932 proteins were retained for subsequent analysis. A scale-free network was constructed with a soft threshold power of 8, and module classification was performed with the parameters of minimum module size = 30 and module cuttree height = 0.3, resulting in the identification of 19 distinct modules. A module-trait relationship was constructed based on the CK, *B. cinerea* treatment, and *A. alternata* treatment groups, and the blue module, which showed the highest correlation with the *A. alternata* treatment, was selected for further analysis. CtChi19 was identified as the core correlated protein in this module with the indices of Absolute Module Membership (abs_MM) = 0.9714 and Absolute Gene Significance (abs_GS) = 0.6091. Taking CtChi19 as the core node, a co-expression network based on the proteomic data was constructed using Cytoscape (Shannon et al., 2003).

For the transcriptomic analysis, WGCNA was performed on the FPKM values of the safflower transcriptome at 12 hpi, 24 hpi, 48 hpi and 72 hpi post *B. cinerea* inoculation, which were previously published before (Ma et al., 2026). The analytical workflow was consistent with that of the proteomic WGCNA. *CtChi19* was identified as the core correlated gene in the corresponding module with the indices of Absolute Module Membership (abs_MM) = 0.8356 and Absolute Gene Significance (abs_GS) = 0.7727. With *CtChi19* as the core node, a co-expression network based on the transcriptomic data was constructed using Cytoscape (Shannon et al., 2003).

Based on the genome-wide protein sequences of safflower (https://www.scuec.edu.cn/safflower/), Gene Ontology (GO) annotation was performed using InterProScan. The species-specific annotation library for safflower was constructed with the R package AnnotationForge. GO enrichment analysis of differentially abundant proteins (DAPs) was implemented using the clusterProfiler package (Wu et al., 2021a). The Benjamini-Hochberg algorithm was adopted for the adjustment of *p*-values (pAdjustMethod), with a cutoff threshold of 0.05.

In the GO enrichment bubble plots, the x-axis represents the Rich factor, and the enrichment significance was quantified by -log10(padj). The color gradient of the bubbles indicates the adjusted p-value, while the bubble size represents the number of enriched proteins.

For the GO biological process (GO-BP) bubble plots, the x-axis shows the GeneRatio or ProteinRatio value, and the y-axis displays the names of GO terms. The number of enriched proteins is reflected by the bubble size, and the color depth represents the magnitude of the adjusted *p*-value.

### 2.5 Gene family analysis

Sequences were extracted from the safflower reference genome (https://www.scuec.edu.cn/safflower/) and subjected to basic bioinformatics analysis using TBtools (Chen et al., 2023). The sequences of the *Arabidopsis thaliana* GH18 and GH19 gene families were downloaded from TAIR (https://www.arabidopsis.org/), and BLASTP was performed against the safflower genome for homologous sequence search. HMMer V3.4 (http://www.hmmer.org/) was used to search the safflower genome with the PF00182 (GH18) and PF00704 (GH19) hidden Markov models (Prakash et al., 2017). Candidate safflower chitinase family sequences identified through integrated BLASTP and HMM searches were further subjected to conserved domain annotation using the NCBI Conserved Domain Database (CDD) and InterPro. Sequences containing incomplete conserved domains or exhibiting excessive truncation were excluded from further analysis (Finn et al., 2016), and members possessing characteristic domains of the chitinase family were selected and named according to their physical order on different safflower chromosomes. The 2000 bp upstream promoter sequences of the chitinase family members were extracted, and cis-acting regulatory element analysis was performed using PlantCARE (https://bioinformatics.psb.ugent.be/webtools/plantcare/html/) (Lescot et al., 2002).

### 2.6 Vector construction

To construct the *pCAMBIA1302-p35S::CtChi19-GFP* plasmid vector, the purified pCAMBIA1302 vector was first double-digested with the restriction endonucleases *NcoI* and *SpeI* (Takara Co., Ltd, Dalian, China). Meanwhile, the CDS sequence of *CtChi19* without terminator was amplified by PCR using safflower cDNA as the template, and the *NcoI* and *SpeI* restriction sites matching the vector were introduced into the upstream and downstream primers, respectively (primer sequences see **Supplementary Table 11**).

Subsequently, the digested linearized pCAMBIA1302 vector and the *CtChi19* insert fragment were subjected to seamless ligation using the One Step Seamless Cloning Kit (KM602, COOKGENE Co., Ltd, Wuhan, China) in accordance with the manufacturer’s instructions. The ligation product was introduced into *E. coli* DH5α chemically competent cells (WEIDI Co., Ltd, Shanghai, China) via chemical transformation, and the transformed cells were spread on LB solid medium plates containing 50 μg/mL kanamycin, followed by incubation at 37 °C for 8–12 h.

Single colonies were picked for liquid culture expansion and plasmid extraction. The correct insertion of the *CtChi19* fragment and the integrity of its open reading frame (ORF) were verified by PCR amplification with the CtChi19-F and GFP-R primers (**Supplementary Table 11**) and subsequent sequencing of the PCR products.

After confirming the correctness of the recombinant plasmid, it was transformed into *Agrobacterium tumefaciens* GV3101 chemically competent cells (WEIDI Co., Ltd, Shanghai, China) following the manufacturer’s instructions. The transformed GV3101 were spread on LB solid medium plates containing 50 μg/mL kanamycin and 25 μg/mL rifampicin, and incubated at 28 °C for 48–60 h. Positive single colonies were selected and propagated in liquid LB medium, then verified by PCR with the CtChi19-F and GFP-R primers (**Supplementary Table 11**), and the accuracy of the inserted sequence was ultimately confirmed by Sanger sequencing.

### 2.7 Subcellular localization via transient expression in *Nicotiana benthamiana*

The transient expression method in *Nicotiana benthamiana* reported by Wydro et al. ( (Wydro et al., 2006) was adopted with appropriate optimizations for the subcellular localization analysis of the CtChi19 protein. The specific procedures were as follows: *Agrobacterium tumefaciens* GV3101 harboring the recombinant vector *pCAMBIA1302-p35S::CtChi19-GFP* was cultured until the OD_600_ reached 0.5–0.6. Bacterial cells were collected by centrifugation, resuspended in infiltration buffer (10 mM MgCl_2_, 10 mM MES, 150 μM acetosyringone), adjusted to an OD_600_ of 0.8, and incubated at room temperature for 2–3 h to induce the expression of virulence genes.

Subsequently, the bacterial suspension was injected into the abaxial epidermis of young leaves of 4–5-week-old *Nicotiana benthamiana* plants using a needleless syringe. After injection, the plants were first cultured in the dark for 12 h, then transferred to a standard growth environment (12 h light/12 h dark, 22 °C) for an additional 2 days to facilitate the sufficient expression of the GFP fusion protein.

Prior to observation, the abaxial epidermis of the injected area was gently peeled off and spread on a glass slide with sterile water. For plasmolysis experiments, sterile water was replaced with 0.4 M mannitol solution, and the sample was incubated for 10 min under moisturized conditions. Imaging was performed using a laser scanning confocal microscope (LSCM) (Leica Co., Ltd, Germany, Stellaris 5): GFP fluorescence signals were excited with a 488 nm laser, and green fluorescence emission signals were collected within the wavelength range of 500–550 nm.

### 2.8 Safflower genetic transformation

The genetic transformation of safflower in this study was performed based on the method established by Guo et al (Guo et al., 2017). with appropriate optimizations. Specifically, safflower plants at the early flowering stage (approximately 8–10 weeks old) were selected as the recipient materials. *Agrobacterium tumefaciens* GV3101 harboring the recombinant vector *pCAMBIA1302-p35S::CtChi19-GFP* was cultured until the OD_600_ reached 0.8; bacterial cells were collected by centrifugation and resuspended in an infiltration buffer containing 5% sucrose and 0.02% Silwet-L77. On the first day of anthesis, whole flower buds and filaments were fully immersed in infiltration buffer for at least 1 minute. This infiltration treatment was applied consecutively for two days. The transformed safflower plants were then cultivated under long-day conditions (16 h light/8 h dark, 22 °C) for 4–5 weeks, after which T_0_ transgenic seeds were harvested.

The T_0_ generation seeds were sown and cultured in nutrient soil under the same long-day condition (16 h light/8 h dark, 22 °C). Positive transgenic plants were identified by PCR and continuously propagated until homozygous T_2_ generation positive plants were obtained (**Supplementary Table 1**). The expression level of CtChi19 in the positive transgenic plants was determined by RT-qPCR (**Supplementary Table 1**).

### 2.9 β-1,3-glucanase activity assay

The assay was performed with modifications based on the method described before (Mohammadi and Karr, 2002). Briefly, the enzyme activity was calculated by determining the production rate of reducing sugars, which was measured as the amount of reducing ends generated from laminarin hydrolysis by β-1,3-glucanase using a microplate reader. Specifically, the enzyme activities in the leaves of transgenic and WT plants at 12 h and 24 h post fungal inoculation were determined strictly following the protocol provided with the assay kit (BC0365, Solarbio Co., Ltd, Beijing, China). One-way analysis of variance (one-way ANOVA) was used for the statistical comparison of data among all groups. The experiment was repeated three times, with consistent results obtained across replicates.

### 2.10 Chitinase activity assay

Chitinase activity was determined in the leaves of transgenic and WT plants at 12 h and 24 h post fungal inoculation, strictly following the protocol provided with the assay kit (BC0365, Solarbio Co., Ltd, Beijing, China). Briefly, Chitinase catalyzes the hydrolysis of chitin to produce N-acetylglucosamine; the intermediate compound generated by heating N-acetylglucosamine with alkali further reacts with p-dimethylaminobenzaldehyde to form a colored product, which exhibits a characteristic absorption peak at 585 nm. The rate of increase in absorbance value at this wavelength reflects the chitinase activity. One-way analysis of variance (one-way ANOVA) was used for the statistical comparison of data among all groups. The experiment was repeated three times, with consistent results obtained across replicates.

## 3 Results

### 3.1 Proteomic analysis of the safflower response pattern to fungal infection

**Figure 1:**
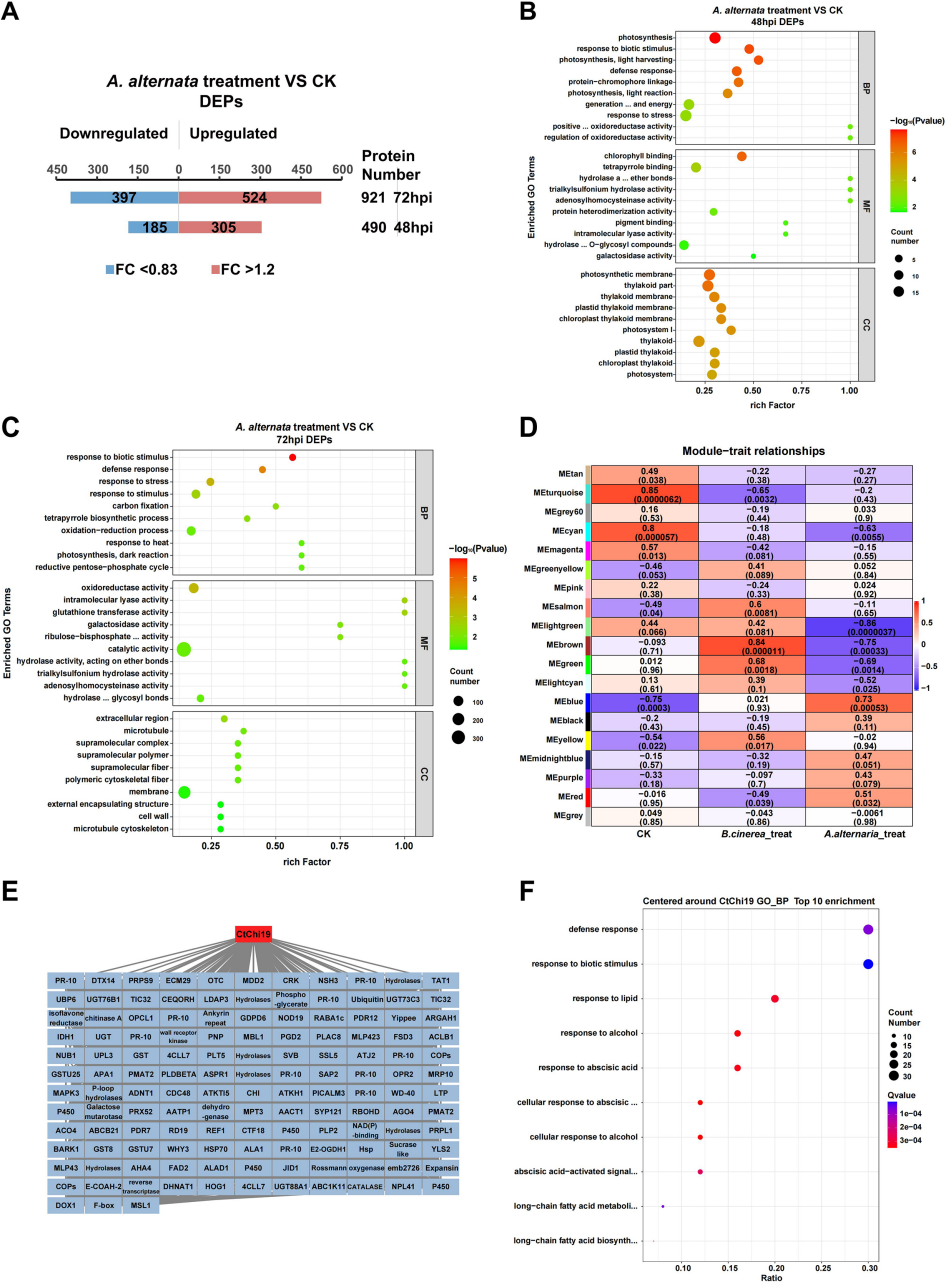
WGCNA analysis of the proteome: **(A)** Proteomic profiling of safflower at 48 hpi and 72 hpi post *A. alternata* inoculation, identifying 490 and 921 differentially expressed proteins (DEPs), respectively. **(B)** GO enrichment analysis of the 490 DEPs at 48 hpi post *A. alternata* inoculation in safflower, revealing significant enrichment of numerous plant immunity-related proteins. **(C)** GO enrichment analysis of the 921 DEPs at 72 hpi post *A. alternata* inoculation in safflower, revealing significant enrichment of numerous plant immunity-related proteins. **(D)** WGCNA clustering of the proteome; all detected proteins with expression levels were clustered into 19 modules. Module-trait relationship analysis was performed for different proteomic modules based on *A. alternata* treatment, *B. cinerea* treatment and control groups, with the blue module showing the strongest correlation with *A. alternata* inoculation. **(E)** Construction of a protein co-expression network centered on CtAH12T0003100 (CtChi19). **(F)** GO biological process (GO_BP) enrichment analysis of proteins co-expressed with CtAH12T0003100 (CtChi19), revealing a strong association of these proteins with plant defense responses.

Proteomic analysis was conducted on safflower leaves following inoculation with *A. alternata* (**Figure 1A**). Briefly, safflower seedlings were subjected to *A. alternat*a inoculation under laboratory conditions, using purified fungal spores for leaf infection. Proteomic sequencing was performed on the treatment and control groups at 48 h and 72 h post-inoculation, respectively. Differentially expressed proteins (DEPs) were defined as those with a fold change ≥ 1.2 or ≤ 0.83 and a *p* < 0.05 (Student’s t-test). The results revealed 490 and 921 differentially expressed proteins (DEPs) at 48 hpi and 72 hpi, respectively (**Figure 1A**; **Supplementary Table 1**). GO enrichment analysis indicated that a large number of plant immunity-related proteins were enriched among the DEGs at both time points (**Figures 1B, C**; **Supplementary Table 2**), demonstrating that this set of proteomic data could reflect the proteomic level changes in safflower in response to *A. alternata* infection.

In the DEPs that were upregulated at the two time points of safflower’s response to *A. alternata*, a large number of proteins related to biotic stress response were identified, including those with direct antibacterial functions such as PR1, PR3, PR5, PR10, Chitinase; proteins associated with systemic acquired resistance (SAR) such as pleiotropic drug resistance (PDR) protein, Barwin-related endoglucanase, methyl esterase and the like; hormone response-related proteins such as mitogen-activated protein kinase (MAPK), glutathione transferase (GST), calreticulin, nicotinamidase; signal transduction-related kinases including Malectin/receptor-like kinase (Malectin/RLK), cysteine-rich RLK, SYR1/PEN1; as well as the cell wall regulatory enzyme pectin methylesterase inhibitor (PMEI) and other such proteins (**Table 1**; **Supplementary Table 1**).

**Table 1:** Typical upregulated & downregulated DEPs at both time points.

Group	DEPs	Description	Group	DEPs	Description
UP	CtAH02T0201200	PR10	DOWN	CtAH04T0194600	ferredoxin/thioredoxin reductase
UP	CtAH02T0201200	PR10	DOWN	CtAH01T0058400	ferredoxin-NADP reductase
UP	CtAH02T0199900	PR10	DOWN	CtAH06T0320000	D-ribulose-5-phosphate-3-epimerase
UP	CtAH02T0200100	PR10	DOWN	CtAH09T0166500	D-ribulose-5-phosphate-3-epimerase
UP	CtAH02T0201100	PR10	DOWN	CtAH04T0029300	photosystem II reaction center
UP	CtAH02T0200000	PR10	DOWN	CtAH11T0103500	ribulose-bisphosphate carboxylase
UP	CtAH02T0199300	PR10	DOWN	CtAH03T0291000	Chlorophyll A-B binding
UP	CtAH02T0202200	PR10	DOWN	CtAH04T0097400	ribulose-bisphosphate carboxylase
UP	CtAH02T0200700	PR10	DOWN	CtAH03T0041700	hypothetical protein
UP	CtAH02T0202100	PR10	DOWN	CtAH12T0157000	phosphoglycerate kinase
UP	CtAH02T0200600	PR10	DOWN	CtAH01T0037300	yloglucan endotransglucosylase/hydrolase
UP	CtAH02T0199700	PR10	DOWN	CtAH11T0022100	yloglucan endotransglucosylase/hydrolase
UP	CtAH03T0179300	Chitinase	DOWN	CtAH10T0112500	alpha/beta-Hydrolases
UP	CtAH03T0178000	Chitinase	DOWN	CtAH03T0153300	beta glucosidase
UP	CtAH08T0250100	Chitinase	DOWN	CtAH03T0154900	beta glucosidase
UP	CtAH03T0178600	chitinase	DOWN	CtAH10T0271100	starch synthase
UP	CtAH12T0003100	chitinase	DOWN	CtAH04T0247300	alpha-glucan water dikinase
UP	CtAH08T0006100	chitinase	DOWN	CtAH03T0154200	beta glucosidase
UP	CtAH12T0171000	chitinase	DOWN	CtAH04T0023200	isocitrate lyase
UP	CtAH05T0085600	PR1	DOWN	CtAH09T0012200	beta galactosidase
UP	CtAH05T0085700	PR1	DOWN	CtAH10T0107700	beta galactosidase
UP	CtAH08T0272400	PR5	DOWN	CtAH05T0009900	beta galactosidase
UP	CtAH04T0201100	GLYCOLATE OXIDASE	DOWN	CtAH11T0245500	Lipoxygenase
UP	CtAH09T0277900	mitogen-activated protein kinase	DOWN	CtAH03T0047700	indole-3-glycerol phosphate synthase
UP	CtAH08T0074300	Kunitz trypsin inhibitor	DOWN	CtAH09T0006000	fatty acid biosynthesis
UP	CtAH10T0221900	oxalyl-CoA synthetase	DOWN	CtAH01T0050500	tryptophan biosynthesis
UP	CtAH12T0008700	Calcium-dependent phosphotriesterase	DOWN	CtAH11T0258000	Adenosylhomocysteinase
UP	CtAH09T0252700	RPM1-interacting protein	DOWN	CtAH11T0186000	Cobalamin-independent synthase
UP	CtAH11T0050600	Malectin/receptor-like kinase	DOWN	CtAH03T0037700	Chalcone-flavanone isomerase
UP	CtAH08T0289500	calreticulin	DOWN	CtAH08T0263800	Histidine biosynthesis
UP	CtAH11T0111800	FAD/NAD(P)-binding oxidoreductase	DOWN	CtAH04T0107500	glycyl-tRNA synthetase
UP	CtAH06T0300300	patatin-like protein	DOWN	CtAH06T0073000	S-adenosyl-L-homocysteine hydrolase
UP	CtAH10T0099000	pectin methylesterase inhibitor	DOWN	CtAH09T0145200	Reactive Intermediate Deaminase
UP	CtAH05T0139400	Cytochrome P450	DOWN	CtAH06T0183000	3-phosphoshikimate 1-carboxyvinyltransferase
UP	CtAH02T0124900	WHIRLY	DOWN	CtAH01T0216100	alpha/beta-Hydrolases
UP	CtAH08T0048800	UDP-Glycosyltransferase	DOWN	CtAH11T0203400	methionine adenosyltransferase
UP	CtAH08T0119800	Glucose/ribitol dehydrogenase			
UP	CtAH09T0017800	pleiotropic drug resistance			
UP	CtAH06T0018400	beta-1,3-glucanase			
UP	CtAH10T0097700	multidrug resistance-associated protein			
UP	CtAH06T0041200	SYR1/PEN1			
UP	CtAH02T0202900	MLP-like protein			
UP	CtAH08T0086800	Glycoside Hydrolase			
UP	CtAH05T0238300	NA			
UP	CtAH02T0200500	MLP-like protein			
UP	CtAH07T0081300	OPC-8:CoA ligase			
UP	CtAH01T0137100	cysteine-rich RLK			
UP	CtAH10T0005600	ammonium transporter			
UP	CtAH05T0238200	Barwin-related endoglucanase			
UP	CtAH02T0203700	MLP-like protein			
UP	CtAH09T0017400	pleiotropic drug resistance			
UP	CtAH02T0021600	Disease resistance-responsive			
UP	CtAH01T0025700	methyl esterase			
UP	CtAH12T0102900	glutathione transferase			
UP	CtAH02T0076200	GEM-RELATED			
UP	CtAH03T0248400	mitochondrial ATPase			
UP	CtAH03T0139600	la-related protein			
UP	CtAH07T0196300	nicotinamidase			
UP	CtAH12T0102600	glutathione transferase			

A set of enzymes associated with photosynthesis and carbohydrate metabolic processes were identified among the proteins downregulated at both time points of safflower’s response to *A. alternata*, including those involved in photosynthesis such as photosystem II reaction center protein, ferredoxin-NADP reductase, D-ribulose-5-phosphate-3-epimerase, ribulose-bisphosphate carboxylase and chlorophyll A-B binding protein; as well as enzymes related to carbohydrate metabolic process, e.g., xyloglucan endotransglucosylase/hydrolase, alpha/beta-hydrolases, beta-glucosidase, starch synthase, beta-galactosidase and others. Additionally, various enzymes participating in the small molecule biosynthetic process were detected, including those involved in fatty acid biosynthesis, tryptophan biosynthesis, histidine biosynthesis, and chalcone-flavanone isomerase, among others(**Table 1**; **Supplementary Table 1**).

Proteomic data from safflower leaves infected by *B. cinerea* were previously documented in a published study (Ma et al., 2026). In the present work, proteomic datasets generated from *A. alternata*-infected safflower were integrated with the publicly available proteomic profiles of *B. cinerea*-infected safflower leaves at 48 and 72 hours post-inoculation (hpi) for weighted gene co-expression network analysis (WGCNA). All detected proteins were clustered into 19 modules (**Figure 1D**; **Supplementary Figure 1B**; **Supplementary Table 3**), among which the Blue module (containing 1179 proteins) exhibited the highest correlation with *A. alternata* infection (correlation coefficient = 0.73, *p* = 0.0053) (**Figure 1D**; **Supplementary Figure 1C**; **Supplementary Table 3**). GO biological process (GO_BP) enrichment analysis of the Blue module identified a large number of defense-related proteins, which were significantly enriched in functional terms such as response to stress, response to biotic stimulus, and response to endogenous stimulus (**Supplementary Figure 1D**; **Supplementary Table 4**). This indicated that the proteins in this module could represent the defense response of safflower against *A. alternata* infection.

Analysis of the gene composition of this module revealed that a safflower chitinase gene, CtAH12T0003100 (later named *CtChi19* following gene family analysis), was located at a key node of the module. A co-expression network centered on this target protein was established (**Figure 1E****;****Supplementary Table 5**). Co-expressed proteins exhibiting high connectivity with CtAH12T0003100 (*CtChi19*) were predominantly enriched in functional terms associated with defense responses and biotic stress adaptation, including defense response, response to biotic stimulus, and response to lipid (**Figure 1F**; **Supplementary Table 6**). Accordingly, on the basis of proteomic profiling results, CtAH12T0003100 (*CtChi19*) was recognized as a key candidate protein mediating safflower resistance to fungal infection and selected for subsequent functional characterization.

### 3.2 Transcriptomic analysis of the safflower response pattern to fungal infection

Weighted gene co-expression network analysis (WGCNA) was performed on previously published transcriptomic datasets from Botrytis cinerea-infected safflower (Ma et al., 2026). Briefly, under laboratory conditions, purified *B. cinerea* spores were used to infect safflower seedling leaves, and transcriptomic sequencing was conducted on the treatment and control groups at 12 h, 24 h, 48 h and 72 h post-inoculation, respectively (Ma et al., 2026).

**Figure 2:**
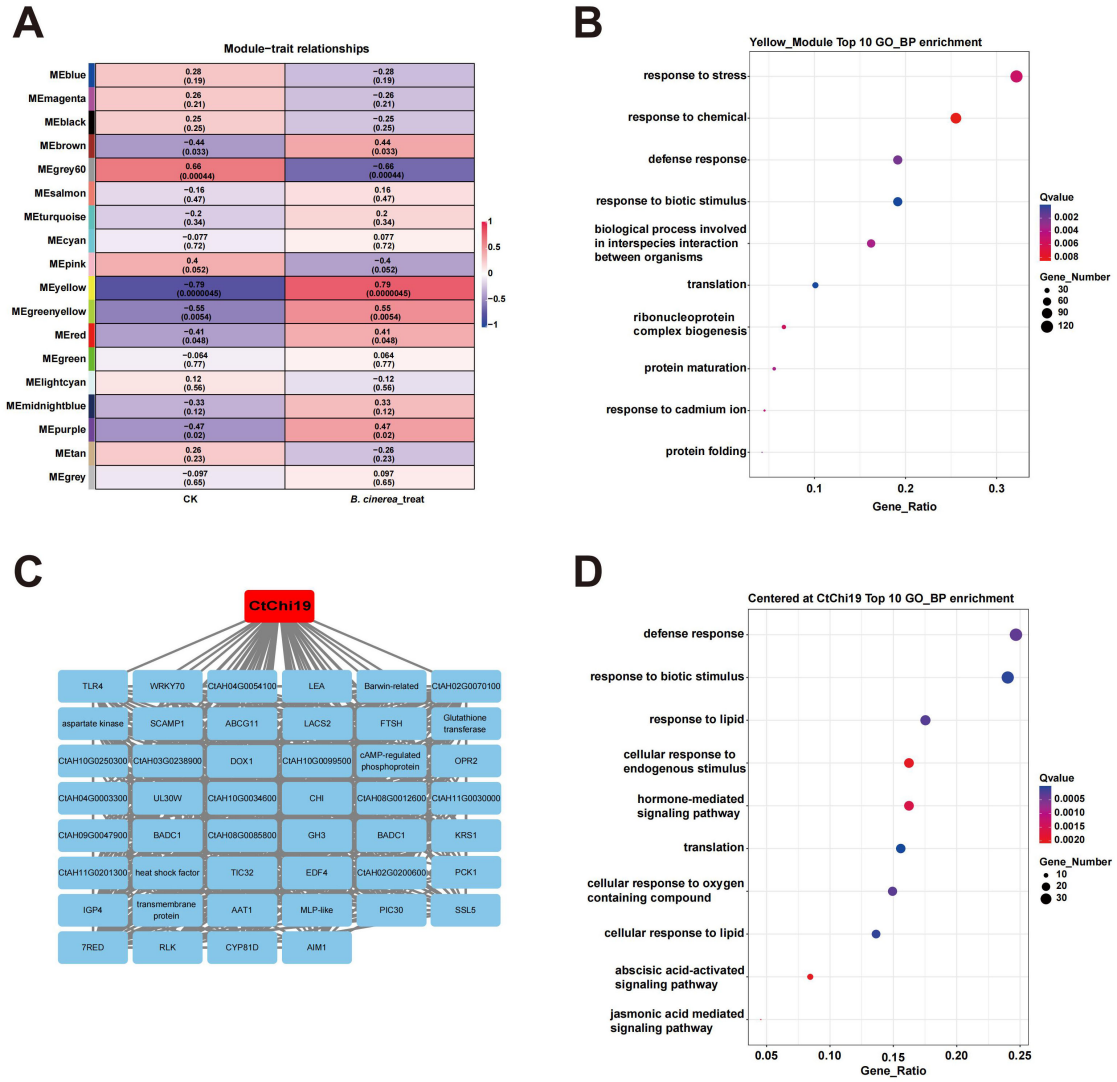
WGCNA analysis of the transcriptome: **(A)** Module-trait relationship analysis of different transcriptomic modules based on *B. cinerea* treatment and control groups, with the yellow module showing the strongest correlation with *B. cinerea* treatment. **(B)** GO biological process (GO_BP) enrichment analysis of the 552 genes in the yellow module (most closely associated with *B. cinerea* inoculation), with most genes enriched in plant disease resistance or defense-related terms. **(C)** Construction of a co-expression network centered on CtAH12T0003100 (*CtChi19*). **(D)** GO biological process (GO_BP) enrichment analysis of the 208 genes co-expressed with CtAH12T0003100 (*CtChi19*), revealing that most of these genes were concentrated in defense or biotic stress response-related terms.

WGCNA was performed on the FPKM (Fragments Per Kilobase of transcript per Million mapped reads) values of all expressed genes in the transcriptome, and the results showed that all detected expressed genes could be clustered into 18 modules (**Figure 2A**; **Supplementary Figure 1E**). Among these modules, the yellow module (containing 552 genes) exhibited the highest correlation with *B. cinerea* infection (correlation coefficient = 0.79) with the most significant statistical significance (*p* = 4.5×10^-6^) (**Figure 2A**). GO enrichment analysis of the yellow module revealed that the top four terms in the biological process (BP) category were enriched in defense-related genes associated with response to stress, response to chemical, defense response and response to biotic stimulus (**Figure 2B**; **Supplementary Table 7**), indicating that this module could represent the defense response of safflower against *B. cinerea* infection.

Analysis of the gene composition within this module revealed that a safflower chitinase gene, CtAH12T0003100 (later named *CtChi19* following gene family analysis), was located at a key node of the module. A co-expression network centered on this target gene was constructed (**Figure 2C**; **Supplementary Table 8**). Functional enrichment analysis revealed that genes co-expressed with CtAH12T0003100 (*CtChi19*) were predominantly enriched in functional terms related to defense and biotic stress responses, including defense response, response to biotic stimulus, and cellular response to endogenous stimulus (**Figure 2D****;****Supplementary Table 9**). Accordingly, based on transcriptomic profiling, CtAH12T0003100 (*CtChi19*) was identified as a critical defense gene mediating safflower responses to fungal invasion. By integrating proteomic and transcriptomic evidence, CtAH12T0003100 (*CtChi19*) was subsequently selected as a pivotal candidate gene for further functional characterization to verify its role in safflower antifungal resistance.

### 3.3 Chitinase gene family in safflower

**Figure 3:**
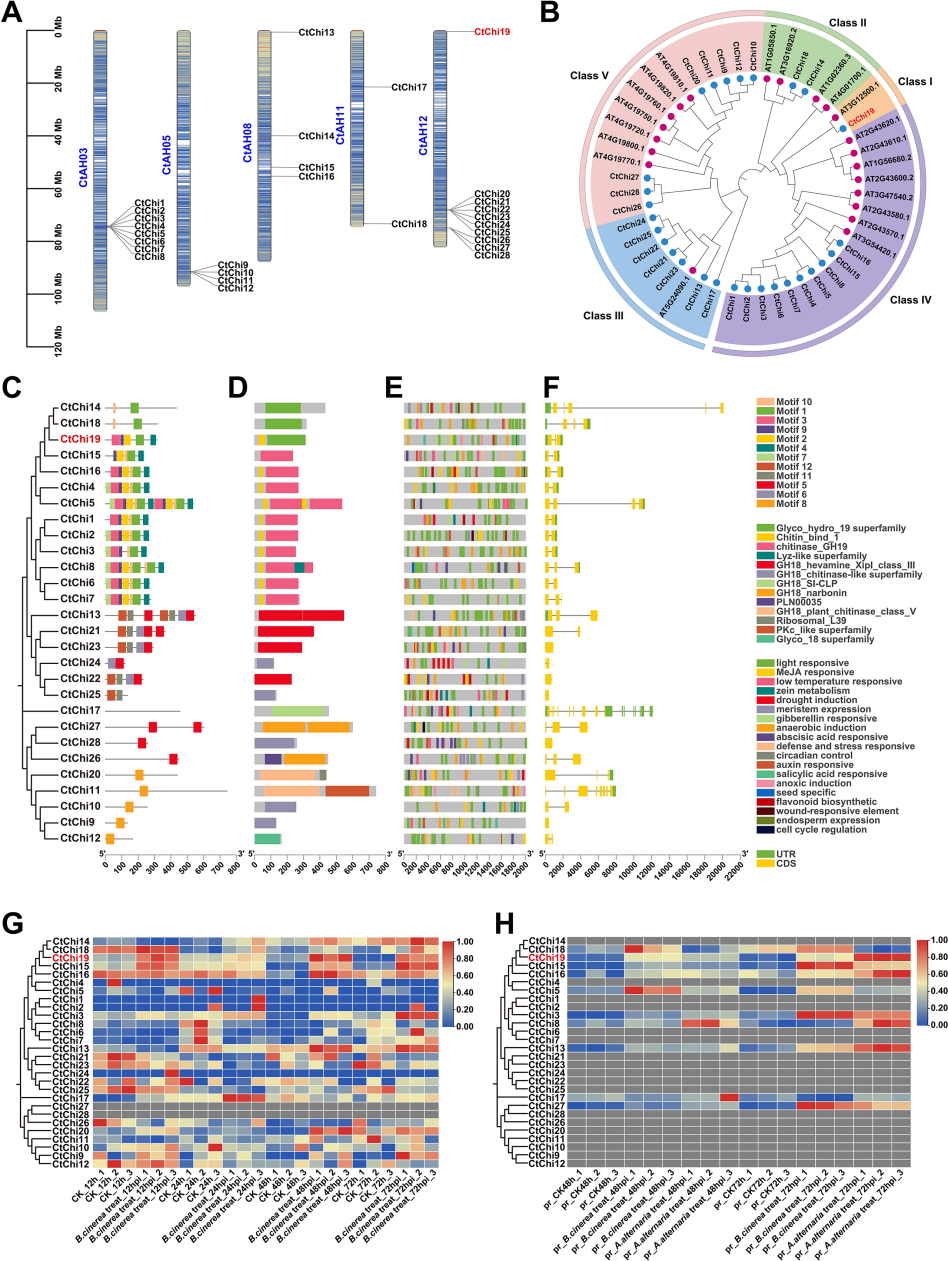
Analysis of the chitinase gene family in safflower: **(A)** Chromosomal distribution of chitinase gene family members in safflower. **(B)** Phylogenetic tree constructed with chitinase gene family members from safflower and *Arabidopsis thaliana*. **(C)** Motif analysis. **(D)** Conserved domain analysis. **(E)** Promoter analysis. **(F)** Gene structure. **(G)** Expression heatmap of chitinase gene family members in safflower at the transcriptomic level. **(H)** Expression heatmap of chitinase gene family members in safflower at the proteomic level.

A comprehensive genome-wide analysis of the chitinase gene family was performed in safflower (**Figure 3**). Using hidden Markov model (HMM) searches and BLASTP analyses, protein sequences across the entire safflower genome were screened. Genes with truncated fragments or incomplete functional domains were excluded, leading to the final identification of 28 chitinase gene family members. The length of the encoded amino acids ranged from 120 to 743 aa, with an average length of 330 aa (**Supplementary Table 10**). Chromosomal localization analysis of the safflower chitinase family members was performed using TBtools software (Chen et al., 2023), revealing that these members are exclusively distributed on chromosomes 3, 5, 8, 11, and 12. According to their physical positions on the chromosomes, these genes were designated as CtChi1 to CtChi28 (**Figure 3A**).

To further clarify their evolutionary relationships, protein sequences of 21 chitinase gene family members from *Arabidopsis thaliana* were downloaded from TAIR, and a phylogenetic tree was constructed together with the 28 safflower chitinase family members (**Figure 3B**; **Supplementary Table 10**). All chitinase members were classified into two glycoside hydrolase families, GH18 and GH19. Based on the Arabidopsis classification criteria and sequence homology, the phylogenetic tree was further divided into five clades: chitinase classes I, II, and IV belong to the GH19 family, while classes III and V belong to the GH18 family (Grover, 2012; Vaghela et al., 2022). Classes IV and V are the larger clades, containing 8 and 7 members from *Arabidopsis* and 10 and 15 members from safflower, respectively. Class III contains 8 members, and class II contains 6 members. Notably, CtChi19 and AT3G12500.1 (basic chitinase, PR-3) were grouped into class I, which are typical defensive enzymes that play crucial roles in plant immune defense against fungal pathogens (Vaghela et al., 2022).

To investigate the functions of the chitinase gene family, the 2 kb sequences upstream of the start codon of CtChi genes were submitted to the PlantCARE website for cis-acting element analysis. The results (**Figure 3E**) showed that the promoter regions of safflower chitinase genes contain multiple cis-elements involved in plant hormone responses, defense responses, and stress responses.

Phylogenetic analysis clustered the CtChi proteins into distinct subgroups. Conserved motif analysis of the CtChi genes (**Figure 3C**) showed that the conserved motifs differed among members of different clades in the phylogenetic tree. Nevertheless, genes from the same evolutionary branch or subgroup shared multiple conserved motifs with similar sequential arrangements. Specifically, *CtChi19* (Class I) contained the unique conserved motifs 3, 9, 2, 1, and 4; Class II members only harbored motifs 10 and 1; the Class IV clade had an extra motif 10 relative to *CtChi19* (Class I); and the Class III clade consisted of motifs 12, 11, 6, and 5. Protein domain prediction (**Figure 3D**) also indicated that most CtChi possessed complete chitinase catalytic domains, with variations in domain length and composition observed across different subgroups.

To explore the structural conservation and diversity of chitinase genes, visualization of the *CtChi* gene structures (**Figure 3F**) revealed that Class III members in the GH18 family contained 1–3 exons. By contrast, *CtChi17* and *CtChi11* in Class IV exhibited a large and dense number of introns, whereas the remaining Class IV members carried 1–3 exons. For GH19 family members, all contained 2–3 exons except *CtChi14*, which harbored 5 exons; among these, *CtChi14, CtChi18*, and *CtChi5* displayed notably long intron lengths. The exon–intron structural patterns were highly conserved for most genes within the same subclade.

Through the integration of transcriptomic and proteomic profiling, the expression patterns of chitinase family genes in response to *B. cinerea* infection were further investigated. At the transcriptomic level (**Figure 3G**), a large number of chitinase genes exhibited dynamic expression changes at different infection time points. Several members, including *CtChi14, CtChi18, CtChi15*, and *CtChi19*, showed significant transcriptional induction after *B. cinerea* treatment, especially at 48 hpi and 72 hpi. However, proteomic analysis (**Figure 3H**) revealed that only a subset of chitinases accumulated detectable protein levels post-infection. In contrast, CtChi19 showed sustained and strong upregulation at both the transcriptional and protein levels, with the highest expression observed at 72 h post-infection with *B. cinerea* and *A. alternata*. This indicates that the *CtChi19* gene may play an important role in the pathogen response process, further supporting its potential as a key functional member of the chitinase gene family in safflower during *B. cinerea* infection.

The results of gene family analysis, transcriptomic heatmaps, and proteomic heatmaps demonstrated that CtChi19 is the chitinase gene in the safflower chitinase family with the strongest response to fungal infection. Consistent with prior transcriptomic and proteomic WGCNA results identifying *CtChi19* as a core hub gene in safflower’s response to fungal infection, *CtChi19* is characterized as a key antifungal defense gene in safflower. This inference was further validated through transgenic experiments in subsequent studies.

### 3.4 Subcellular localization of CtChi19

**Figure 4:**
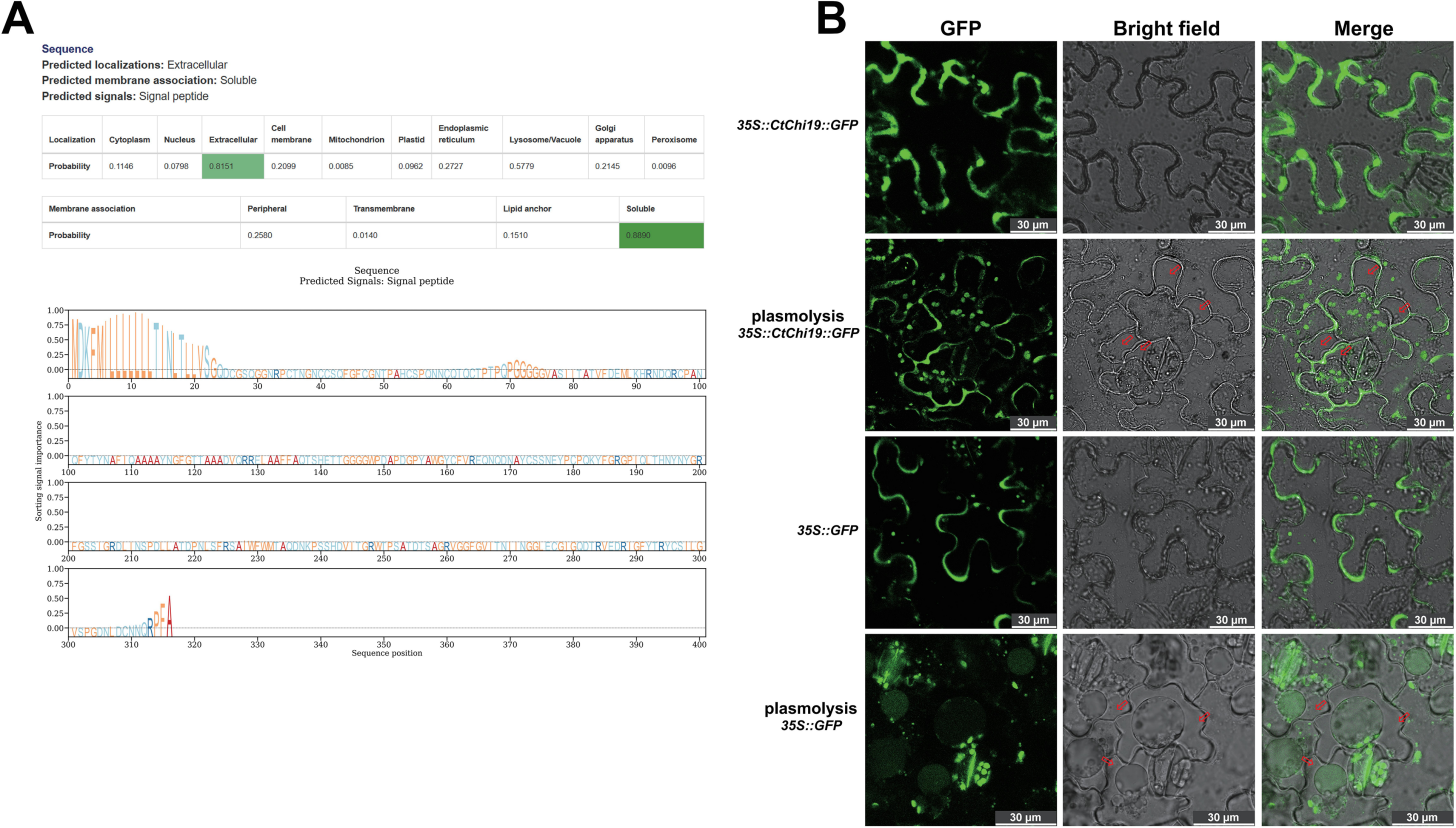
Subcellular localization of CtChi19: **(A)** Subcellular localization prediction of CtChi19 by DeepLoc 2.1; **(B)** The *35S::CtChi19::GFP* vector was constructed, and the green fluorescent signal of the CtChi19-GFP fusion protein was observed via Confocal Laser Scanning Microscopy (CLSM) using the *Nicotiana benthamiana* transient expression system. Subcellular localization of CtChi19 was determined after plasmolysis; the red arrows indicate the gaps formed after cytoplasmic plasmolysis. CtChi19 was found to be distributed in the extracellular space, cell wall, and cytoplasm following plasmolysis. All images are single optical sections acquired via CLSM using a Leica 20×/0.75 DRY objective. Scale Bars: 30 μm.

DeepLoc 2.1 was used to predict the subcellular localization of CtChi19, which was inferred to be localized in the extracellular space (**Figure 4A**) (Ødum et al., 2024). Furthermore, the subcellular localization of CtChi19 was examined via transient expression in Nicotiana benthamiana. Confocal laser scanning microscopy (CLSM) observations (**Figure 4B**) demonstrated that CtChi19 localized to the extracellular space, cell wall and cytoplasm following plasmolysis. This confirms that CtChi19 can be secreted into the apoplast, consistent with the bioinformatics prediction results. Such a localization pattern of CtChi19 is consistent with its plant immune function of degrading the cell walls of invading fungi.

### 3.5 Transgenic validation of *CtChi19* enhancing safflower resistance to *B. cinerea* and *A. alternata*

To further explore the contribution of *CtChi19* to safflower disease resistance, transgenic safflower plants overexpressing *CtChi19* were initially generated. The CDS fragment of *CtChi19* without terminator was amplified from safflower cDNA, and the overexpression recombinant vector *pCAMBIA1302-p35S::CtChi19-GFP* was constructed using the pCAMBIA1302 vector (**Supplementary Figure 2A**). The recombinant plasmid was transformed into *E. coli* DH5α chemically competent cells, and single colonies were picked for PCR identification and sequencing (**Supplementary Figure 2B**). After confirming the sequencing accuracy, the positive single colony was expanded in culture to extract the plasmid, which was then transformed into *Agrobacterium tumefaciens* GV3101 chemically competent cells. Single colonies were selected, and positive clones were verified by PCR (**Supplementary Figure 2C**).

**Figure 5:**
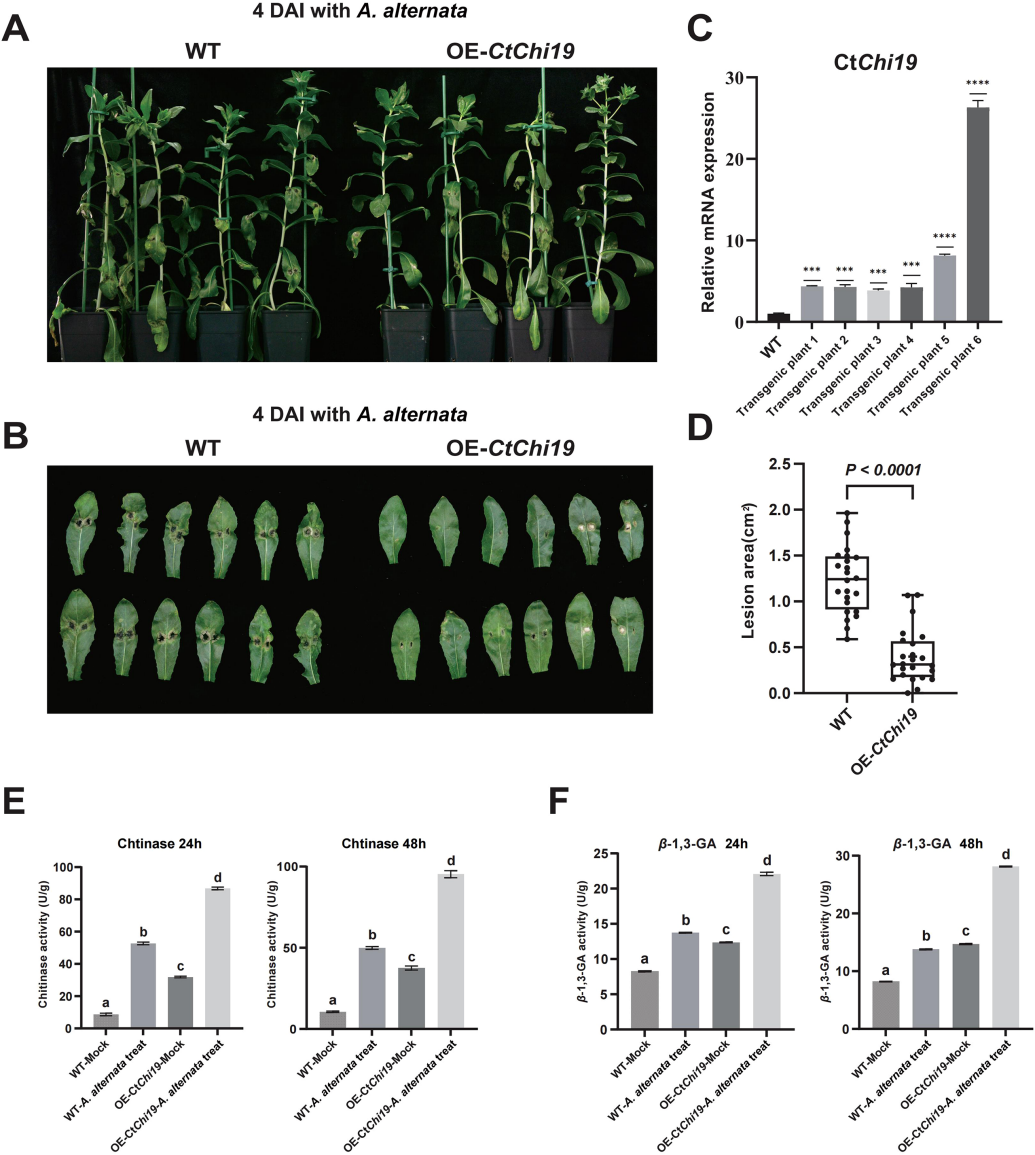
Transgenic validation of *CtChi19*-mediated enhanced resistance to *A. alternata*: **(A)** and **(B)** Inoculation of safflower seedlings with *A. alternata* mycelia, including 6 plants each of OE-CtChi19 transgenic plants and WT plants;**(C)** RT-qPCR analysis confirmed that the *CtChi19* was upregulated in all 6 OE-*CtChi19* transgenic plants used for the infection assay, compared with the wild-type plants;**(D)** Lesion areas on leaves were measured using ImageJ at 4 days post-inoculation. The lesion areas of OE-*CtChi19* plants were significantly smaller than those of WT plants, indicating that overexpression of *CtChi19* significantly enhances safflower resistance to *A. alternata*. This experiment was repeated three times with consistent results. **(E)** Chitinase activity in leaf tissues was determined at 24 h and 48 h post-inoculation, comparing inoculated and mock-treated groups of WT and OE-*CtChi19* transgenic plants. Mixed samples from 6 plants were used for enzyme activity detection in each group. This experiment was repeated three times with consistent results. **(F)** β-1,3-glucanase activity in leaf tissues was determined at 24 h and 48 h post-inoculation, comparing inoculated and mock-treated groups of WT and OE-*CtChi19* transgenic plants. Mixed samples from 6 plants were used for enzyme activity detection in each group. This experiment was repeated three times with consistent results. ***p < 0.001; ****p < 0.0001.

Safflower cultivar YH-7 was infiltrated with the recombinant *Agrobacterium*, and T_2_ generation *p35S::CtChi19-GFP* transgenic safflower plants were obtained. PCR identification verified the successful genetic transformation (**Supplementary Figure 2D**). Reverse transcription quantitative real-time PCR (RT-qPCR) confirmed the overexpression of the *CtChi19* gene in transgenic safflower lines (**Figure 5C**). The mycelia of *Alternaria alternata* were inoculated on the leaves of T_2_-generation OE-*CtChi19* transgenic safflower plants, followed by incubation under moisturized conditions (**Figures 5A, B**). The lesion area of infected leaves was quantified using ImageJ software at 4 days post inoculation (4 dpi). Compared with wild-type (WT) plants, OE-*CtChi19* transgenic plants exhibited an extremely significant reduction in leaf damage caused by *A. alternata* infection (*p* < 0.0001) (**Figure 5D**), indicating remarkably enhanced resistance to *A. alternata* in transgenic safflower. These results demonstrate that overexpression of CtChi19 can improve the disease resistance of safflower against *A. alternata*.

Chitinase and β-1,3-glucanase activities were further quantified in wild-type (WT) and transgenic safflower plants. The results showed that chitinase activity in OE-*CtChi19* transgenic plants was significantly higher than that in WT plants under mock conditions, demonstrating that overexpression of *CtChi19* in safflower can significantly enhance chitinase activity. In WT safflower, *A. alternata* infection increased chitinase activity, indicating that chitinase is an important defensive response of plants to biotic stress. Chitinase activity in OE-*CtChi19* transgenic plants was significantly higher than that in WT plants, and the induction of chitinase activity by *A. alternata* infection was more pronounced in OE-*CtChi19* plants (**Figure 5E**).

Additionally, some pathogenesis-related (PR) proteins possess β-1,3-glucanase activity (e.g., PR-2, PR-5) (Wang et al., 2021). Plant β-1,3-glucanase can act synergistically with chitinase to degrade the cell walls of invading pathogens by hydrolyzing β-1,3-glucan and chitin (Amian et al., 2011; Dos Santos and Franco, 2023). Therefore, to assess the responsiveness of OE-*CtChi19* transgenic plants to A. alternata, β-1,3-glucanase activities were compared between wild-type (WT) and OE-*CtChi19* transgenic lines. Overexpression of *CtChi19* in safflower significantly enhanced β-1,3-glucanase activity, suggesting a synergistic effect between chitinase and β-1,3-glucanase in plants. After *A. alternata* infection, the β-1,3-glucanase activity in OE-*CtChi19* transgenic plants increased more drastically compared with the mock-treated WT group (**Figure 5F**). The relative transcript abundance of the *PR5* gene, which encodes a protein exhibiting β-1,3-glucanase activity, was also examined. The expression profile of *PR5* was found to be largely consistent with the dynamic changes in β-1,3-glucanase activity. (**Supplementary Figures 3A, B**).

**Figure 6:**
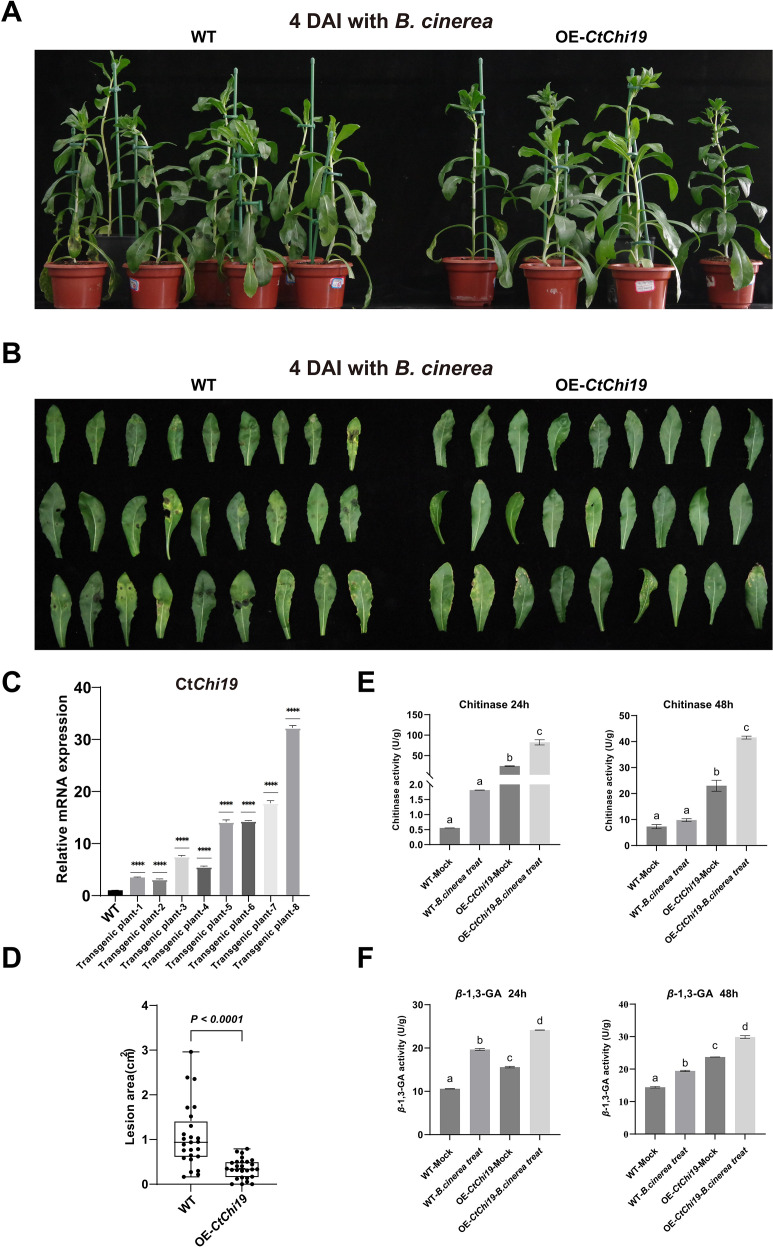
Transgenic validation of *CtChi19*-mediated enhanced resistance to *B. cinerea*: **(A)** and **(B)** Inoculation of safflower seedlings with *B. cinerea* mycelia, including 8 plants each of OE-*CtChi19* transgenic lines and WT plants; **(C)** RT-qPCR analysis confirmed that the *CtChi19* was upregulated in all 8 OE-Ct*Chi19* transgenic plants used for the infection assay compared with WT plants; **(D)** Lesion areas on leaves were measured using ImageJ at 4 days post-inoculation. The lesion areas of OE-*CtChi19* plants were significantly smaller than those of WT plants, indicating that overexpression of *CtChi19* significantly enhances safflower resistance to *B. cinerea*. This experiment was repeated three times with consistent results; **(E)** Chitinase activity in leaf tissues was determined at 24 h and 48 h post-inoculation, comparing inoculated and mock-treated groups of WT and OE-*CtChi19* transgenic plants. Mixed samples from 8 plants were used for enzyme activity detection in each group. This experiment was repeated three times with consistent results; **(F)** β-1,3-glucanase activity in leaf tissues was determined at 24 h and 48 h post-inoculation, comparing inoculated and mock-treated groups of WT and OE-*CtChi19* transgenic plants. Mixed samples from 8 plants were used for enzyme activity detection in each group. This experiment was repeated three times with consistent results. ****p < 0.0001.

The resistance of OE-*CtChi19* transgenic safflower plants to Botrytis cinerea was further validated. RT-qPCR analysis confirmed that CtChi19 was significantly overexpressed in the transgenic lines employed for pathogen inoculation (**Figure 6C**). Subsequently, T_2_-generation OE-*CtChi19* transgenic safflower plants were inoculated with *B. cinerea* mycelia (**Figures 6A, B**), and the leaf lesion area was quantified using ImageJ software at 4 days post inoculation (4 dpi).Compared with wild-type (WT) plants, OE-*CtChi19* transgenic plants showed an extremely significant reduction in leaf damage caused by *B. cinerea* infection (*p* < 0.0001) (**Figure 6D**), indicating that transgenic safflower exhibited remarkably enhanced resistance to *B. cinerea*. These results confirmed that overexpression of *CtChi19* can improve the resistance of safflower to *B. cinerea*. Subsequently, chitinase and β-1,3-glucanase activities were quantified in WT and transgenic safflower lines. The results revealed that overexpression of *CtChi19* significantly elevated both enzymatic activities in transgenic plants; moreover, these activities were further induced and upregulated following *B. cinerea* inoculation. (**Figures 6E, F**). The relative transcript levels of the *PR5* gene, which encodes a protein with β-1,3-glucanase activity, were also analyzed. The expression profile of *PR5* was largely consistent with the dynamic fluctuations in β-1,3-glucanase activity (**Supplementary Figures 3C, D**).

All the above results demonstrate that overexpression of *CtChi19* in safflower can enhance its resistance to infection by both *B. cinerea* and *A. alternata*.

## 4 Discussion

Safflower (*Carthamus tinctorius*), an economic crop of the Asteraceae family with both medicinal and oil-producing values, is widely cultivated worldwide. Its seeds are rich in linoleic acid and linolenic acid, and are regarded as a healthy source of edible oil (Fan et al., 2023). Its bright flowers can not only be used as a natural dye, but also contain a variety of medicinal active ingredients such as Hydroxysafflor yellow A (HSYA), which have been developed for pharmaceutical preparations (Furuya et al., 1987). However, safflower is highly susceptible to fungal infection during cultivation, which often leads to yield reduction or quality degradation (Víchová et al., 2011; Singh et al., 2019). At present, research on safflower diseases mainly focuses on field disease investigation and pathogen identification, while molecular research on its disease resistance mechanism is still extremely scarce. This knowledge gap constrains the in-depth elucidation of safflower disease resistance mechanisms and impedes the efficient breeding of disease-resistant cultivars.

To identify candidate disease response-related proteins in safflower, safflower plants were inoculated with *A. alternata*, the causal agent of safflower leaf spot disease, and proteomic analyses were performed at 48 and 72 hours post-inoculation (hpi). Upon *A. alternata* infection, safflower exhibited significant upregulation of numerous defense-related proteins, such as PR1, PR5, PR10, CtChi19, PMEI and so on, while the expression of proteins associated with photosynthesis and growth metabolism was downregulated, such as photosystem II reaction center protein, starch synthase, histidine biosynthesis and so on (**Table 1**; **Supplementary Table 1**). This expression pattern aligns with the “Growth-Defense Tradeoff” hypothesis in plant-pathogen interactions (Huot et al., 2014; Karasov et al., 2017). This resource allocation strategy represents an adaptive mechanism forged during the long-term evolution of plants. Previous studies have validated that the activation of defense pathways is often accompanied by the transcriptional repression of growth-related genes. Notably, this growth–defense trade-off is predominantly regulated in a programmed manner by the ADR1-mediated transcriptional network, rather than merely stemming from resource competition (Hu et al., 2026). Similarly, coordinated expression patterns featuring the upregulation of defense genes and the downregulation of growth-related metabolism have been documented in potato, Arabidopsis, and other plant species under pathogen stress (Zrimec et al., 2025). These findings provide further support for the universality of this trade-off mechanism in plant defense responses.

Integrated with previously published transcriptomic and proteomic datasets (Ma et al., 2026), WGCNA was performed to identify a subset of genes exhibiting the highest contribution to safflower defense responses against fungal infection (**Supplementary Tables 5**, **S8**). The majority of these genes are functionally associated with plant immunity (**Supplementary Figure 1D**; **Supplementary Table 4**; **Figure 2B**; **Supplementary Table 7**), thus providing a foundational gene resource for safflower disease-resistance breeding. Subsequently, *CtChi19*, a core hub gene among these candidates, was selected for systematic gene family analysis, and its disease-resistance function was verified via overexpression in safflower.

Analysis of the safflower chitinase gene family showed that a phylogenetic tree was constructed using 21 chitinase gene family members from *Arabidopsis thaliana* (GH18 and GH19 families; download link: https://www.arabidopsis.org/browse/gene_family/GlycosideHydrolase) and 28 chitinase gene family members identified in safflower. Based on their domains and functions, the safflower chitinase gene family can be classified into 5 classes (Grover, 2012; Vaghela et al., 2022). These members are exclusively distributed on chromosomes 3, 5, 8, 11, and 12 (**Figure 3A**), with some existing in tandem arrangements on chromosomes 3 and 12. Specifically, the 8 tandem members on chromosome 3 all belong to Class IV, the 4 tandem members on chromosome 5 belong to Class V, and the two clusters of tandem members on chromosome 12 belong to Class V and Class III, respectively (**Figure 3B**).

The clustered members of the safflower chitinase gene family share similar gene structures, domains, and promoters (**Figures 3C–F**), suggesting that these genes may have originated from tandem gene duplication. Tandemly duplicated genes can perform similar functions and are located at the same chromosomal positions, facilitating simultaneous activation and rapid production of large quantities of functionally similar products in a short period (Leister, 2004).

The remaining non-clustered gene family members include *CtChi13*, *CtChi14*, *CtChi15*, and *CtChi16* on chromosome 8, as well as *CtChi17* and *CtChi18* on chromosome 11, which are distributed across Class II, Class III, and Class IV. Notably, *CtChi19* on chromosome 12 is the only Class I member in the safflower chitinase gene family (**Figure 3A**). Further transcriptomic and proteomic heatmap analyses (**Figures 3G, H**) revealed that the four clusters of clustered gene family members show weak responses to *B. cinerea* and *A. alternata* infection, while non-clustered members exhibit strong responses. For instance, *CtChi15*, *CtChi16*, *CtChi17*, *CtChi18*, and *CtChi19* all show significant induction, with *CtChi19* displaying the strongest response to both *B. cinerea* and *A. alternata* (**Figures 3G, H**). Integrated with prior WGCNA analyses of transcriptomic and proteomic datasets, *CtChi19* is identified as the most critical chitinase gene within the safflower chitinase family in defense against fungal infection.

In terms of promoter response elements, most members of the safflower chitinase gene family contain cis-acting elements involved in biotic stress responses, including methyl jasmonate-responsive elements, salicylic acid-responsive elements, defense and stress-responsive elements, and wound-responsive elements. Among these, the jasmonic acid (JA) and salicylic acid (SA) pathways are core components of plant disease resistance signaling (Pieterse et al., 2012), suggesting that genes harboring these responsive elements may function in defending against pathogens. Additionally, hormone-responsive elements (e.g., auxin, gibberellin, and abscisic acid-responsive elements) are present, indicating that these genes are subject to extensive hormonal regulation and may participate in the crosstalk between growth and defense signaling pathways (Waadt et al., 2022).

The presence of cis-acting elements related to abiotic stress responses (including low-temperature-responsive elements, drought-inducible elements, and anaerobic-inducible elements) in the promoters of safflower Chi family members suggests that chitinase genes carrying these elements may contribute to the regulation of plant environmental adaptability. For example, Ltchi7 can be induced by drought and salt stresses (Tapia et al., 2011). *Arabidopsis* plants overexpressing barley *HvCh*i show enhanced drought tolerance by elevating the activities of oxidative protective enzymes (Wan et al., 2024). In addition, chitinases exert positive protective roles in soybean under salt and heavy metal stresses (Mészáros et al., 2014). In *Arabidopsis*, chitinases participate in the tolerance to multiple abiotic stresses including low temperature, freezing, high temperature, and strong light (Takenaka et al., 2009).

Meanwhile, cis-elements associated with plant growth and development are also distributed in the promoters of safflower Chi family members, such as meristem expression elements, seed-specific expression elements, endosperm expression elements, and cell cycle regulatory elements. For instance, *Arabidopsis AtCTL1* plays a vital role in plant growth and development, with persistent functions in cell wall biosynthesis and cell elongation. Its activity is modulated by environmental stimuli, thereby reshaping organ morphology (Hermans et al., 2010). Mutations in chitinase-like genes lead to ectopic lignin deposition, abnormal cell morphology, and excessive ethylene production (Zhong et al., 2002). In terms of environmental signal perception, light-responsive elements and circadian rhythm regulatory elements are present in the promoters of these genes, suggesting that their expression may be modulated by photoperiod. With respect to plant metabolic regulation, the corresponding genes harbor regulatory elements associated with tryptophan metabolism and flavonoid biosynthesis. In support of this, studies in grape have documented that flavonoids can bind to chitinases, implying that flavonoids may participate in mediating specific physiological functions of chitinases (Filippi et al., 2016). All these results indicate that members of the safflower chitinase gene family may also be involved in the growth and developmental processes of safflower.

In summary, the above results systematically reveal that the promoters of safflower *CtChi* genes integrate multiple regulatory signals including hormones, stresses, and growth/developmental cues. Genes of this family are widely implicated in regulating plant growth and development, environmental adaptability, and stress tolerance by responding to diverse endogenous and exogenous signals.

Transient expression of *CtChi19* in *Nicotiana benthamiana* demonstrated that this gene is distributed in the extracellular space, cell wall, and cytoplasm. The ability of *CtChi19* to be secreted into the extracellular space is associated with its role in combating fungal colonization in the apoplast (**Figure 4B**). Furthermore, overexpression of *CtChi19* in safflower confirmed that this gene enhances safflower resistance to *A. alternata* (**Figure 5**) and *B. cinerea* (**Figure 6**).

Extensive studies have demonstrated that overexpression of chitinase genes across diverse plant species enhances host resistance to a broad spectrum of fungal pathogens. For instance, heterologous overexpression of chitinase genes from tobacco or rice in peanut (*Arachis hypogaea*) enhanced its resistance to *Cercospora arachidicola* (the causal agent of peanut leaf spot disease or Tikka disease) (Rohini and Sankara Rao, 2001; Iqbal et al., 2012). Transgenic cucumber plants expressing a rice chitinase gene showed increased resistance to *B. cinerea* (Tabei et al., 1998). In strawberry, overexpression of the chitinase gene ch5B from common bean (*Phaseolus vulgaris*) improved resistance to *B. cinerea*, and this resistance was directly correlated with the presence of the heterologous CH5B protein and a significant increase in leaf chitinase activity (Vellicce et al., 2006). Overexpression of *MdCHI1* in apple (Malus×domestica) enhanced resistance to the fungal pathogens *Colletotrichum gloeosporioides* and *A alternata* compared to wild-type apples, with elevated chitinase activity being a key contributor to the enhanced resistance (Wang et al., 2021). In pepper (*Capsicum annuum*), VIGS-mediated silencing of the *CaChiIII7* gene markedly compromised host resistance to *Colletotrichum acutatum*, as manifested by enlarged lesion areas, enhanced fungal proliferation, downregulated expression of defense-related genes, and diminished H_2_O_2_ accumulation and cell death. Conversely, transient ectopic overexpression of *CaChiIII7* strengthened pepper resistance to *C. acutatum*, as reflected by the upregulated transcription of defense genes, elevated H_2_O_2_ production, and intensified hypersensitive cell death, thereby restricting pathogen proliferation and spread (Ali et al., 2020). In soybean (*Glycine max*), overexpression of the chitinase gene *CmCH1* from the biocontrol fungus *Coniothyrium minitans* enhanced transgenic plant resistance to *Sclerotinia sclerotiorum* without affecting soybean growth, development, or agronomic traits (Yang et al., 2020).

Overexpression of the rice chitinase gene LOC_Os11g47510 (highly expressed in the disease-resistant cultivar Tetep) in the susceptible cultivar TP309 significantly enhanced the resistance of transgenic TP309 plants to *Rhizoctonia solani*, with the resistance level showing a positive correlation with the expression level of the transgene (Richa et al., 2017). Similarly, transferring the maize-derived chitinase 1 gene (*Chit1*) into rice resulted in a significant reduction in the severity of rice blast (*Magnaporthe oryzae*) symptoms (Anwaar et al., 2024).

Heterologous expression of *LcCHI2*, a chitinase gene derived from *Leymus chinensis* (a grass species with exceptional drought and salt-alkali tolerance), conferred simultaneous enhancements in disease resistance and salt stress tolerance in transgenic tobacco and maize. Importantly, this genetic modification did not compromise the growth or yield of these transgenic lines under standard cultivation conditions (Liu et al., 2020), thereby offering a promising strategy for improving both salt-alkali stress tolerance and pathogen resistance in dicot and monocot crops alike.

Collectively, these cases demonstrate that overexpression of either homologous or heterologous chitinase genes can effectively enhance plant defense against specific fungal pathogens, indicating that the disease resistance function of chitinases is highly conserved across plant species.

## 5 Conclusion

Necrotrophic fungi *A. alternata* and *B. cinerea* cause severe yield losses in safflower, yet the molecular immune responses of safflower to these pathogens remain unclear and no breeding-suitable resistance genes have been identified to date. In this study, safflower plants were inoculated with *A. alternata*, and proteomic profiling was conducted at 48 and 72 hours post-inoculation (hpi). By integrating these datasets with previously published transcriptomic and proteomic profiles of *B. cinerea*-infected safflower (Ma et al., 2026), candidate resistance genes associated with fungal infection were identified via WGCNA, with the hub chitinase gene *CtChi19* selected as the primary research target. Systematic analysis of the safflower chitinase family showed *CtChi19* is the earliest and most significantly induced member upon *B. cinerea* infection, and subcellular localization confirmed its extracellular/cell wall localization. Stable transgenic safflower lines overexpressing *CtChi19* were generated, and these lines were confirmed to exhibit significantly enhanced resistance to both *B. cinerea* and *A. alternata* This study not only deepens researchers’ understanding of the molecular mechanisms underlying safflower disease resistance, but also identifies a panel of resistance candidates responsive to *B. cinerea* and *A. alternata*, and validates *CtChi19* as a key gene that enhances safflower antifungal resistance. In doing so, it provides valuable genetic resources and a theoretical basis for safflower resistance breeding, lays a foundation for in-depth investigation into the molecular mechanisms of safflower antifungal defense, and supports the breeding and selection of disease-resistant safflower cultivars.

## Data Availability

The datasets presented in this study can be found in online repositories. The names of the repository/repositories and accession number(s) can be found in the article/**Supplementary Material**.
