# Supplementary material for: CtChi19: a valuable gene for improving resistance to Botrytis cinerea and Alternaria alternata in Carthamus tinctorius
Source: Front Plant Sci. 2026 Apr 24;17:1797378. doi: 10.3389/fpls.2026.1797378 (PMC13153071; doi:10.3389/fpls.2026.1797378)
Supplement: Supplementary file 12 [file DataSheet12.docx]

Supplementary Material

Supplementary file for

***CtChi19*: A Valuable Gene for Improving Resistance to *Botrytis cinerea* and *Alternaria alternata* in Carthamus tinctorius**

Kang Ma^1+^, Xiaoyan Wang^1+^, Kangjun Fan^1^, Kehui Zhang^1^, Lu Lv^1^, Zhaojun Wei^2^, Jiao Liu^1^, Hong Liu^1^, Jian Wei^3*^, & Rui Qin^1*^

Kang Ma & Xiaoyan Wang contributed equally to this work.

1Hubei Provincial Key Laboratory for Protection and Application of Special Plant Germplasm in Wuling Area of China, College of Life Sciences, South-Central Minzu University, Wuhan 430074, China.

2School of Biological Science and Engineering, North Minzu University, Yinchuan 750021, China

3Institute for Safflower Industry Research of Shihezi University, Shihezi 832003, China.

* Correspondence: Rui Qin & Jian Wei
Rui Qin: [qinrui@scuec.edu.cn](mailto:qinrui@scuec.edu.cn)

Jian Wei: weijian@jlau.edu.cn

Keywords: Carthamus tinctorius; Fungal Resistance; Chitinase; Plant Pathogen Interaction; plant immunity


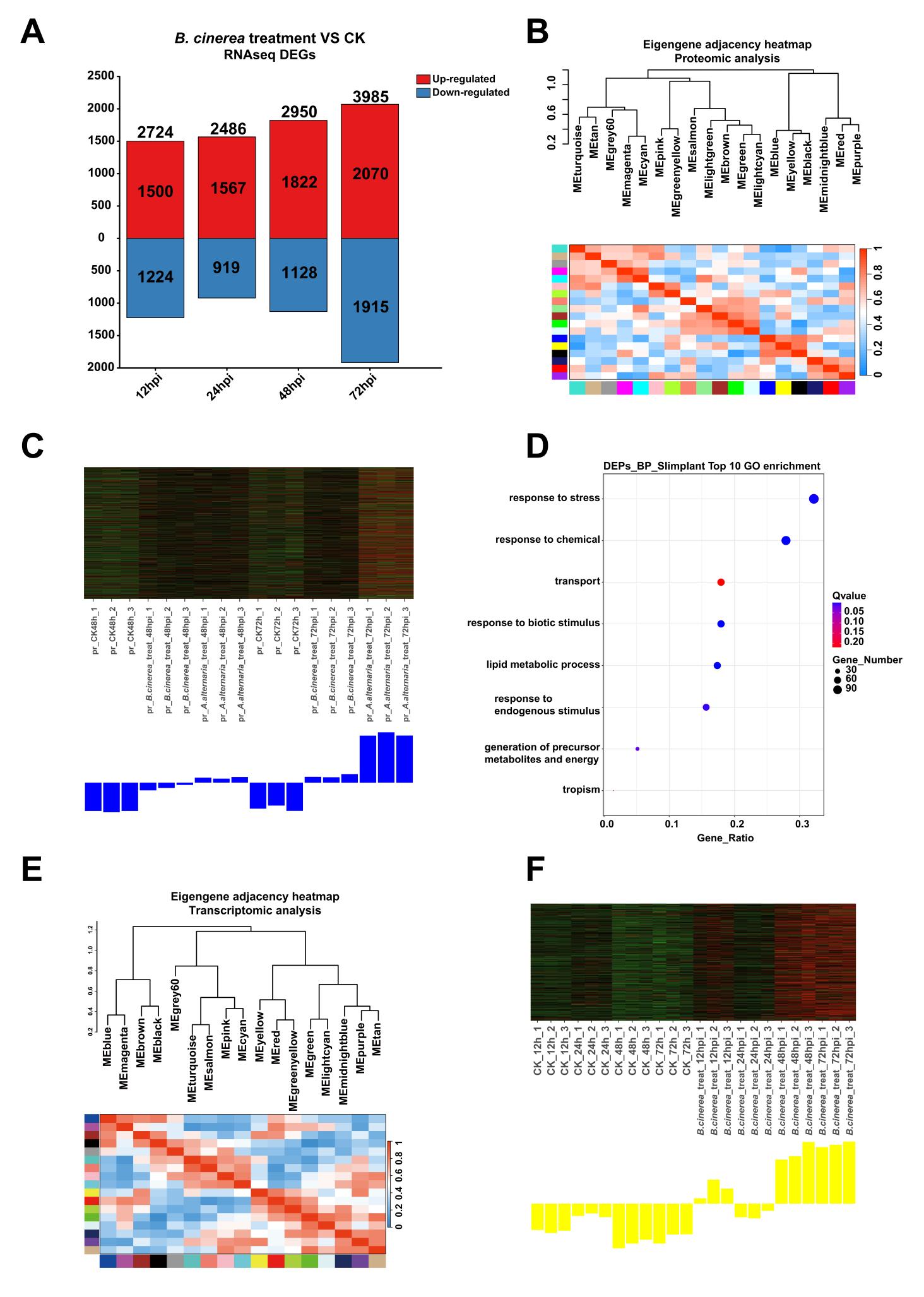


**Figure S1 WGCNA analysis of the proteome and transcriptome (related to Fig. 1 and Fig. 2):**

(A) Transcriptomic profiling of safflower upon *B. cinerea* inoculation showing an increase in the number of differentially expressed genes (DEGs) with the progression of infection time.(B) WGCNA analysis was performed by integrating the proteomic data of safflower infected by *A. alternata* and that infected by *B. cinerea*; all detected proteins were clustered into 19 modules.(C) Blue module *A. alternata*-treat module eigengene (ME) and gene heatmap; proteomic WGCNA analysis revealed the blue module had the strongest correlation with *A. alternata* inoculation.(D) GO biological process (GO_BP) enrichment analysis of the 481 differentially expressed proteins (DEPs) in the blue module (most closely associated with *A. alternata* inoculation), revealing significant enrichment of numerous disease resistance-related proteins.(E) WGCNA analysis of the transcriptomic data of safflower infected by *B. cinerea*; all detected genes with expression levels were classified into 18 modules.(F) Yellow module *B. cinerea*-treat module eigengene (ME) and gene heatmap; transcriptomic WGCNA analysis revealed the yellow module had the strongest correlation with *B. cinerea* inoculation.


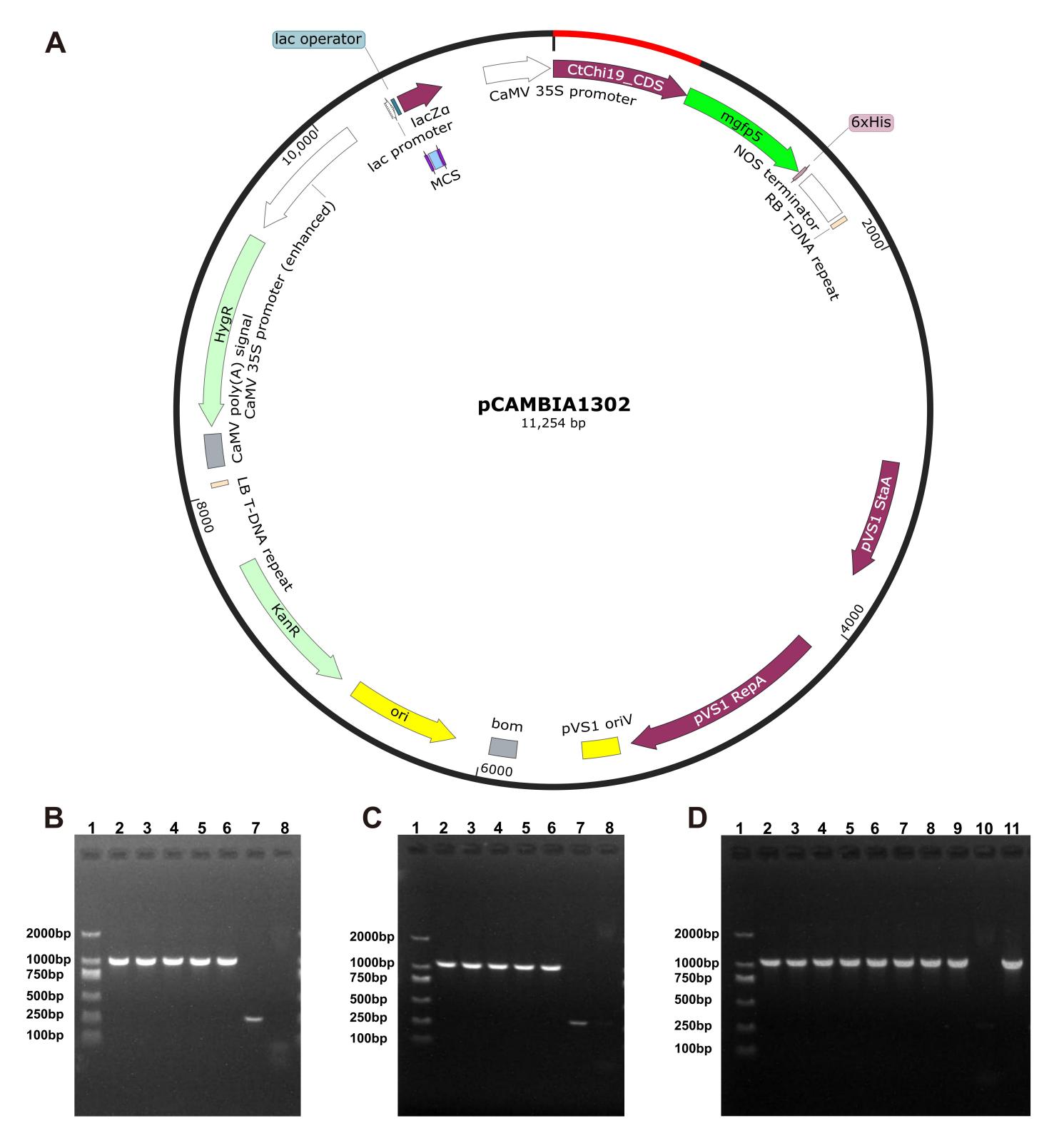


**Figure S2 Construction of OE-CtChi19 vector and identification of transgenic plants:**

(A) The CtChi19 CDS sequence with the terminator removed was inserted between the p35S promoter and GFP on the basis of the pCAMBIA1302 vector.(B) Detection of recombinant 35S::CtChi19::GFP positive plasmids by PCR in E. coli DH5α. Lane 1: DNA marker; Lanes 2–6: Single E. coli colonies transformed with recombinant 35S::CtChi19::GFP plasmids; Lane 7: Non-recombinant plasmid; Lane 8: Negative control.(C) Detection of recombinant 35S::CtChi19::GFP positive plasmids by PCR in Agrobacterium tumefaciens GV3101. Lane 1: DNA marker; Lanes 2–6: Single A. tumefaciens colonies transformed with recombinant 35S::CtChi19::GFP plasmids; Lane 7: Non-recombinant plasmid; Lane 8: Negative control.(D) Detection of the 35S::CtChi19::GFP recombinant fragment in transgenic plants by PCR. Lane 1: DNA marker; Lanes 2–9: OE-CtChi19 transgenic plants; Lane 10: Negative control; Lane 11: Positive control.


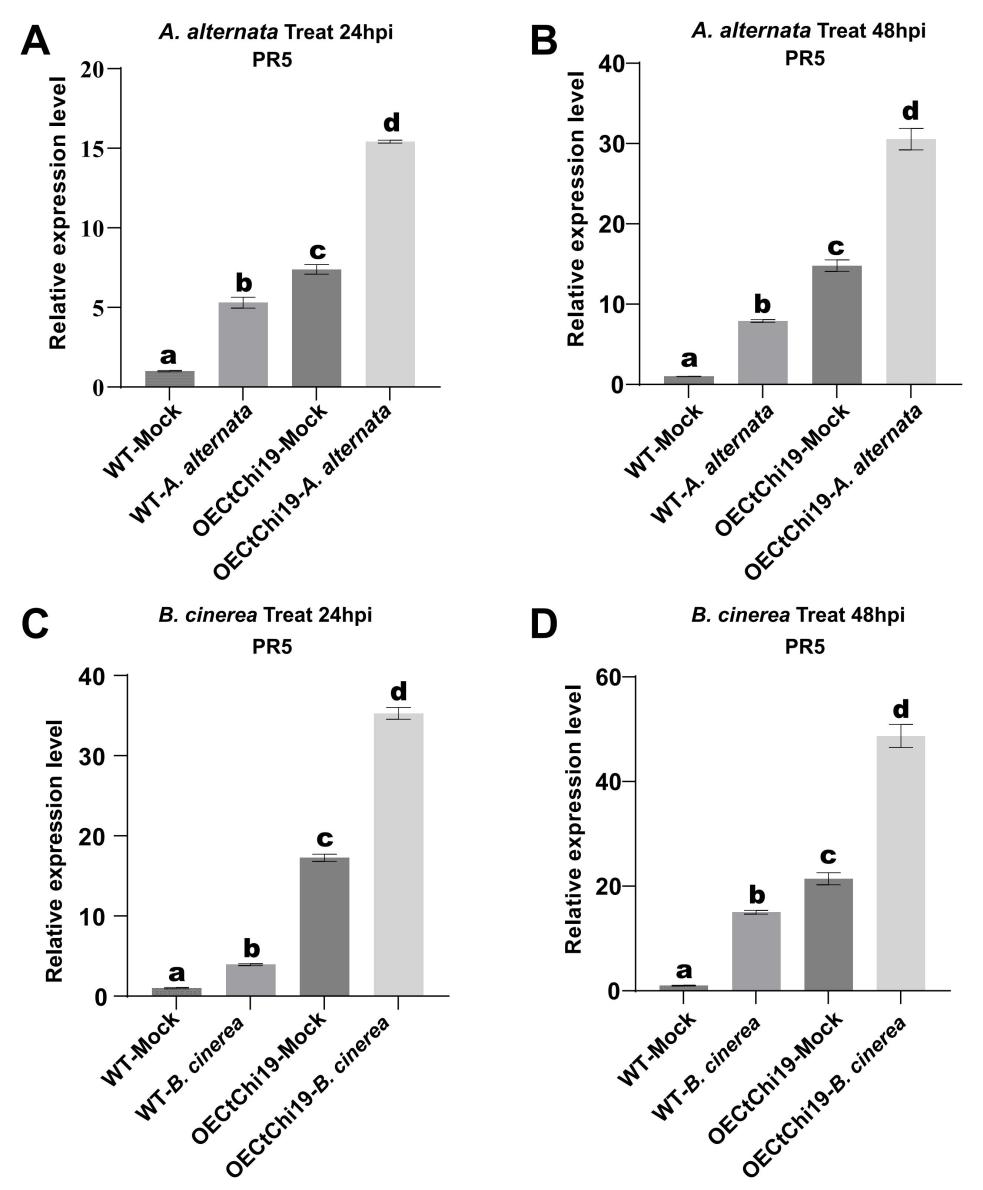


**Figure S3 Characterization of the CtChi19 overexpression plant in response to fungal infection.**

Transcript levels of the PR5 gene in wild-type (WT) and *CtChi19* overexpression (OE) plants at 24 (A) and 48 (B) hours post-inoculation (hpi) with *A. alternata*. Similar expression patterns of PR5 were observed in independent experiments conducted at 24hpi (C) and 48hpi (D) with *B. cinerea*, confirming the enhanced defense response in the OE plants. Relative expression was normalized to the internal control gene and presented as mean ± SD (n = 3). Different letters indicate significant differences between groups (one-way ANOVA, P < 0.05).
